# Low Serum Levels of Zinc, Selenium, and Vitamin D3 Are Biomarkers of Airway Inflammation and Poor Asthma Control: A Two-Centre Study

**DOI:** 10.7759/cureus.41082

**Published:** 2023-06-28

**Authors:** Shetanshu Srivastava, Vandana Tiwari, Shivani Singh, Ritu Karoli, Piyali Bhattacharya, Nikhil Gupta

**Affiliations:** 1 Pediatrics, Dr. Ram Manohar Lohia Institute of Medical Sciences, Lucknow, IND; 2 Biochemistry, Dr. Ram Manohar Lohia Institute of Medical Sciences, Lucknow, IND; 3 Medicine, Dr. Ram Manohar Lohia Institute of Medical Sciences, Lucknow, IND; 4 Pediatrics, Sanjay Gandhi Post Graduate Institute of Medical Sciences, Lucknow, IND

**Keywords:** magnesium, s, vitamin d3, serum zinc, trace elements, asthma control

## Abstract

Background

Asthma is a chronic inflammatory disease with its control being affected by underlying oxidative stress. Trace elements, along with vitamin D3, play an important role in immune alterations leading to an imbalance of Th1/Th2 helper cells. However, their role in asthma pathogenesis and control is inconsistent and inconclusive. The objective of our study was to assess levels of serum trace elements like zinc, copper, selenium, iron, magnesium, vitamin D3 levels, IgE, and HsCRP in asthmatic children, compare with healthy controls, and assess their association with the level of asthma control.

Methods

A cross-sectional study was conducted from 2019 to 2021 enrolling 100 asthmatic children and 75 healthy controls. The level of asthma control was assessed as uncontrolled, partly controlled, and controlled asthma as per GINA Guidelines. Mean and standard deviation were calculated for each element and mean differences between groups were analyzed by student t-test. A p-value of <0.05 was considered significant.

Results

The mean age was 8.75±2.89 yrs in cases and 9.04±2.79 in controls. A total of 57.6% of cases had atopic comorbidities. The mean serum zinc levels were 51±12.8 mg/dl, which was very low in asthmatic children as compared to 60±18.2mg/dl (p-value 0.0002) in healthy controls. Serum selenium was 13±3 µg/dl in asthmatics vs. 15±4 µg/dl (p-value 0.0002) in healthy controls. Serum copper was 115.2±21.92µg/dl vs. 125.3±31.99µg/dl (p-value 0.015), Serum vitamin D3 levels were 13.07±7.82ng/ml vs. 17.82±14.62 ng/ml(p-value 0.006) in both groups, respectively. SIgE and HsCRP were high in asthmatic children suggestive of eosinophilic inflammation. Serum zinc was 49±5.45 mg/dl in the uncontrolled group, 53±6.1 in the partly controlled, and 58±8.0 in the well-controlled group (p<0.0001). Serum selenium was 10± 3.0 µg/dl in the uncontrolled group vs. 13± 2.0 and 14± 2.0 µg/dl in the partly controlled and well-controlled groups, respectively (p-value <0.0001). Vitamin D3 was significantly low (9.32±5.95ng/dl) in the uncontrolled group vs. 12.99±4.97 and 13.40±5.92 ng/dl(p<0.005) in the partly controlled and well-controlled groups respectively. Vitamin D3 showed a strong positive correlation with zinc (r=0.4,p< 0.0001) and a negative correlation with inflammatory markers like SIgE and HsCRP.

Conclusion

Children with asthma had low zinc, selenium, and vitamin D3 levels, and were associated with airway inflammation and poor asthma control.

## Introduction

Asthma is a chronic inflammatory disorder of the airways characterized by variable bronchial obstruction and hyper-responsiveness [1]. It is a multifactorial disease with interactions between genetic susceptibility, host factors, and environmental exposures. There is a growing focus on the exploration of attributable risk factors that may affect asthma control and severity. Nearly 300 million people have asthma annually, causing an economic burden globally [2]. Poor asthma control can worsen life quality of life [3]. The increase in the prevalence of bronchial asthma in children needs to be explored. It is known that inflammation, oxidative stress, and immune dysregulation lead to asthma progression [4]. Evidence suggests that oxidative stress and inflammation leading to an imbalance of Th1/Th2 helper cells play critical roles in the initiation and development of asthma [5]. This also correlates with disease severity and control. Asthmatic children have been found to have significantly increased serum oxidative stress and decreased antioxidant enzymatic activity. Essential trace elements such as zinc (Zn), copper (Cu) and selenium (Se), iron (Fe), and magnesium (Mg) are known to play essential roles in immune system function, and their alterations may result in higher inflammatory responses and oxidative stress. However, correlations have not been examined between these trace elements with oxidative stress, inflammation, immune system response, and lung function in asthmatic children and their alleged role in asthma control.

Serum IgE and HsCRP are known to be involved in the inflammatory cascade in asthma [6,7]. Studies have explored the associations between trace elements and respiratory health, but results are inconsistent [8]. Deficiency in 25-hydroxy vitamin D3 is reported to increase lung airway reactivity. It is a potent immunological modulator and may affect asthma severity and control [9]. However, the effect of trace elements coupled with vitamin D3 level has not been explored in asthma assessment, and reports are conflicting in children; hence, we planned this study. In the present study, serum levels of trace elements like zinc, copper, selenium, iron, magnesium, Vitamin D3, hs-CRP, and IgE were assessed in asthmatic children, their association with asthma control, and compared with healthy controls. Knowledge of these biochemical risk factors may pave way to novel management and therapeutic options. 

## Materials and methods

Selection of participants

A cross-sectional intramural-funded, two-center study was conducted from 2019 to 2021. Dr. Ram Manohar Lohia Institute of Medical Sciences (RMLIMS), a tertiary referral center, conducted the study by recruiting subjects referred from the Sanjay Gandhi Post Graduate Institute of Medical Sciences hospital with asthma. All the biochemical tests were done at the Department of Biochemistry RMLIMS after taking ethical consent from the Institutional research committee dated 15.10.2019, IEC NO 23/19.

Newly diagnosed patients with asthma aged 5 -15 yrs were enrolled as cases, and age and sex-matched individuals were included as healthy controls. Asthma was diagnosed based on history, examination, and spirometry findings. The control level was assessed as uncontrolled, partly controlled, and controlled asthma as per GINA Guidelines [3]. Simple random sampling was done and the sample size was calculated using the formula N= Z2p(1-p)/L2. Here, N = No. of Subjects Z = Confidence interval, an std. the value, which is 1.96 p = Estimated proportion of Population (Prevalence) L= Margin level (±5 %).

With a 5% possibility of drop out of samples, a total of 175 subjects were enrolled. One hundred cases with bronchial asthma were enrolled from the asthma clinic and 75 healthy age and sex-matched children were enrolled as controls after taking parental consent. Children with severe malnutrition, refusal of consent, and a history of zinc, vitamin D, iron, oral or inhaled steroid intake in the past two weeks were excluded from the study. Baseline data like age, gender, anthropometric parameters, comorbidities, and parental history of asthma and allergy were collected. A total of 5 ml of venous blood samples were obtained for biochemical estimation. Zinc, selenium, and copper were estimated by using Inductively Coupled Plasma Mass Spectrophotometry (ICPMS) [10]. Serum magnesium, iron, and IgE, HsCRP levels were analyzed using commercial reagents on an automated Beckman Coulter analyzer [11]. Serum 25 hydroxy Vitamin D3 was estimated by the electrochemiluminescence method.

Data analysis

Statistical Package for the Social Sciences SPSS (version 21.0, Chicago, IL, USA) was used to analyze all data. Mean and standard deviation (SD) were calculated for each element. Frequencies in categorical data were assessed by the Chi-square test. The differences between the asthma group and the control group were compared with an independent t-test mean differences between the two groups. ANOVA test was used to analyze the correlation between serum trace element and level of asthma control and to ascertain whether the difference between these groups was statistically significant (P < 0.05).

## Results

One hundred asthmatic cases and 75 healthy controls were enrolled in the study. Among the cases, 39(39.0%) were with uncontrolled asthma, 53(53.0%) were with partly-controlled asthma, and 8(8.0%) were with well-controlled asthma. Baseline characteristics like age, gender, and Body mass index were comparable in both groups, as shown in Table 1.

**Table 1:** Demographic and baseline characteristics of study subjects

	Total (n=175)	Asthmatic Cases (n=100)	Healthy Controls (n=75)	Student t-test	
Variables				‘t’	‘p’
Age(years) Mean±SD (range 5-15)		8.75±2.89	9.04±2.79	1.592	0.113
Weight (kg)		26.11±8.70	28.44±8.15	1.805	0.073
Height(cm)		127.92±16.54	129.63±14.51	0.711	0.478
BMI (kg/m^2^)		15.95±1.37	16.64±3.84	1.663	0.0982
Family history of asthma	33	26 (26.0)	7(9.3)		
Atopic Comorbidities Absent	111	36 (36.0)	75(100.0)	75.676	<0.001
Atopic Comorbidities Present	64	64 (57.6)	0		
Allergic rhinitis		48 (48.0)			
Allergic conjunctivitis		12(12%)			
Atopic dermatitis		12(12%)			

The mean age among both groups was 8.75 ± 2.89 years vs. 9.04 ± 2.79 years (p=0.113). Mean weight, height, and body mass index were comparable in both groups. The male:female ratio was 2:1 in both groups. Family history of asthma was present in 26(26.0%) in the case group vs. 7(9.3%) (p-value 0.005) in the control group. Atopic comorbidities were present in 64 (57.6%) asthmatic cases, out of which 48 (48%) had allergic rhinitis, 12(12%)had allergic conjunctivitis, and 12(12%) had atopic dermatitis.

Trace elements and vitamin D levels in the asthma case group and healthy controls are shown in Table 2. Serum zinc levels were 51 ± 12.8 mg/dl in the asthma case group vs. 60 ± 18.2 mg/dl in the healthy controls (p-value 0.0002); selenium levels were 13 ± 3.0 µg/dl in the asthma case group vs. 15±4.0 µg/dl in the healthy controls (p-value 0.0002), which were very low in case of the group as compared to controls and difference was highly significant. Serum magnesium was 2.10 mg/dl ± 0.25 vs. 2.04 ± 0.28 mg/dl (value 0.117) in both groups, which was within the normal range. Copper levels were 115.2±21.92 µg/dl among asthmatic children vs. 125.25 ± 31.99 µg/dl in controls (p-value 0.015) and iron was 50.0 ± 33.37 µg/dl among asthmatic children vs. 55 ± 33.12 µg/dl in controls (p-value 0.326) which was comparable among groups. Among both groups, serum Vitamin D3 levels were 13.07 ± 7.82 ng/ml in the asthma case group vs. 17.82 ± 14.62 ng/ml in the healthy controls (p-value 0.006), serum IgE was 1014.48 ± 732.11 in the asthma case group vs. 347.26 ± 563.62 IU/ml in the healthy controls (p-value<0.001). However, HsCRP levels were significantly higher in the asthma group (0.006 ± 0.004 mg/ml) vs. in the healthy controls (0.004 ± 0.003 mg/ml) (p-value 0.0004).

**Table 2:** Trace elements and vitamin D levels among cases and healthy controls

		Cases (n=100)		Controls (n=75)		Student ‘t’ test	
	Normal value	Mean	SD	Mean	SD	‘t’	‘p’
Zinc (mg/dl)	60-110	51	12.8	60	18.2	3.840	0.0002
Magnesium (mg/dl)	1.8- 2.6	2.10	0.25	2.04	0.28	1.576	0.117
Selenium (µg/dl)	12.0-16	13	3.0	15	4.0	3.781	0.0002*
Copper (µg/dl)	70-140	115.22	21.92	125.25	31.99	2.460	0.015
Iron (µg/dl)	50-150	50.0	33.37	55.97	33.12	0.984	0.326
Vitamin D3 (ng/ml)	>30	13.07	7.82	17.82	14.62	-2.762	0.006
IgE (IU/ml)	50-87	1014.48	732.11	347.26	563.62	6.566	<0.001
Hs-CRP (mg/ml)	<0.003	0.006	0.004	0.004	0.003	-3.631	0.0004

Table 3 shows a comparison of biochemical parameters with the level of asthma control. Serum zinc levels were 49 ± 5.45mg/dl in the uncontrolled group vs. 53 ± 6.1mg/dl in the partly-controlled and 58 ± 8.0 mg/dl in the well-controlled asthma group (p <0.0001). Serum selenium levels were 0.10 ± 0.03µg/dl in the uncontrolled group, 0.13±0.02µg/dl in the partly controlled, and 0.14 ± 0.02 µg/dl in the well-controlled asthma group (p-value <0.0001). Vitamin D3 was significantly low in uncontrolled group at 9.32 ± 5.95 ng/ml, versus 12.99 ± 4.97ng/ml in the partly-controlled, and 13.40 ± 5.92 ng/ml in the well-controlled group (p 0.005). Serum iron was significantly different with levels at 51.92 ± 34.29 vs 53.75 ± 29.37 vs 87.63 ± 41.05 µg/dl in the uncontrolled, partly-controlled, and well-controlled groups, respectively (p-value 0.017). Magnesium was 2.12 ± 0.33 mg/dl, 2.09 ± 0.18 mg/dl, and 2.12 ± 0.20 mg/dl (p 0.846) in the uncontrolled, partly-controlled, and well-controlled groups, respectively; copper levels were not significantly different at 117 ± 23.70 µg/dl, 114.22 ± 20.64 µg/dl, and 109.51 ± 22.37 µg/dl in the uncontrolled, partly-controlled, and well-controlled groups, respectively (p 0.562). Serum IgE was 1515.0 ± 644.0 IU/ml in the uncontrolled asthma group, 747.0 ± 616.17 in the partly-controlled, and 341.86 ± 229.36IU/ml in the well-controlled group (p<0.001). Hs-CRP was significantly higher in the uncontrolled asthma group at 0.005 ± 0.004 mg/ml compared to 0.002 ± 0.003 mg/ml and 0.002 ± 0.005 mg/ml partly-controlled, and well-controlled groups (p-value=0.002) among groups.

**Table 3:** Association of trace elements and Vitamin D3 with the level of asthma control

Parameters	Uncontrolled (n=39)		Partly controlled (n=53)		Well controlled (n=8)		ANOVA	
	Mean	SD	Mean	SD	Mean	SD	F	‘p’
Zinc (mg/dl)	49	5.45	53	6.1	58	8	9.522	<0.0001
Magnesium (mg/dl)	2.12	0.33	2.09	0.18	2.12	0.2	0.167	0.846
Copper ()µg/dl)	117.74	23.7	114.22	20.64	109.51	22.37	0.579	0.562
Selenium ()µg/dl)	10	3	13	2	14	2	20.154	<0.0001
Iron ()µg/dl)	51.92	34.29	53.75	29.37	87.63	41.05	4.265	0.017
Vitamin D (ng/ml)	9.32	5.95	12.99	4.97	13.4	5.92	5.585	0.005
IgE (IU/ml)	1515.14	644.44	747.6	616.17	341.86	229.36	23.201	<0.001
Hs-CRP (mg/ml)	0.005	0.004	0.002	0.003	0.002	0.005	6.689	0.002

As shown in Table 4, vitamin D3 has a significant positive correlation with zinc (r=0.4, p< 0.001), a weak correlation with copper (r=0.15, p 0.042), and a negative correlation with hs-CRP and S IgE (r=-0.19, p=0.01 and r=-0.26, p 0.001 respectively). Hs-CRP was significantly negatively correlated with zinc (r=-0.25, p<0.0001), whereas copper and selenium showed a weak positive correlation with hs-CRP (r=0.28, p<0.0001 and r=0.13, p=0.09) respectively.

**Table 4:** Correlation of Vitamin D level with IgE and hsCRP levels

Variables	Hs-CRP (mg/ml)	IgE (IU/ml)	Iron (µg/dl)	Zinc (mg/dl)	Copper (µg/dl)	Selenium (µg/dl)
Vitamin D3 (ng/ml)	r=-0.19 p=0.01*	r=-0.26 p=0.001*	r=-0.47 p=0.53	r=0.4 P<0.001*	r=0.15 p=0.042*	r=0.04 p=0.85
Hs-CRP (mg/L)			r=-0.41 p=0.58	r=-0.25 p<0.0001*	r=0.28 p<0.0001*	r=0.13 p=0.09

## Discussion

In our study, the levels of the trace elements zinc and selenium were significantly low in asthmatic children as compared to healthy controls. Asthma is an airway inflammatory disease; trace elements may have a role in the inflammatory process.

Similar studies [12-16] have shown significantly low levels of zinc and other trace elements like selenium; however, the results are inconsistent with some [17,18]. The reason could be due to differences in inclusion criteria like the age group, method of assessment, and sample size. Moreover, studies showing normal zinc levels have recruited asthmatics already on inhaled steroids which may also affect zinc levels. [17]. A review by Song Mao et al. in 2018 [8] concludes that alterations of trace elements may be a biomarker of asthma risk, and studies should be performed to prove their role in asthma. A negative relationship between serum zinc levels and total IgE [14] has been reported though we did not find it in our study. Vitamin D3 levels and zinc had a significant positive correlation in our study.

A recent review states overproduction of reactive oxygen species (ROS) has been implicated in the development of asthma [18 ]. Thus, more studies are warranted to investigate the complicated interactions between ROS and different types of antioxidants for the restoration of the redox balance under pathologic conditions. Though we did not study the mechanistic approach, we collected clinical data and found an association of trace elements with the level of asthma control.

Monitoring and managing zinc levels may be a new therapeutic modality in asthmatic patients with low vitamin D3. Selenium acts by inhibiting reactive oxygen species (ROS) production and chronic inflammation. We found that asthma cases had a lower level of selenium than controls, which may be because it increased the immune response to allergens by increasing T-helper responses [19]. Low selenium was also associated with uncontrolled asthma, as in our study. Copper affects the activation of inflammatory mediators, inflammatory cell recruitment, immune cell function, and airway remodeling; thus, it may have a role in the progression of asthma. Copper deficiency and excess can induce oxidative stress and chronic inflammation [20]. Copper levels in our study were in the normal range, though lower in the asthmatic group. Interestingly, the levels were higher in the uncontrolled group than in the partly-controlled and well-controlled groups. This difference was statistically significant, indicating a role of copper excess in oxidative stress. Magnesium relaxes bronchial smooth muscles and airways and is a treatment modality during acute asthma flare-ups. Studies in adults report low serum magnesium levels [21]. However, in our study, asthmatic children had normal serum magnesium levels, as reported in some studies [22]; magnesium is mainly an intracellular ion, and normal serum Mg levels may not reflect intracellular magnesium deficiency. Therefore, the estimation of intracellular magnesium concentration may be more accurate [23].

On the other hand, iron is a critical element in many oxidative reactions. Only free iron takes part in the inflammatory process producing free radicals. Our findings showed that asthmatic patients had serum iron levels on the lower side of the normal range as in other studies [24,25]. We did not assess the levels of transport proteins like transferrin which is an indicator of iron status. A review [26] states that iron, plays a key role in the pathogenesis and severity of asthma, and levels not of iron, but also of iron-stabilizing proteins are needed for asthma control. Vitamin D insufficiency was present in all study subjects, but levels were significantly lower in asthmatic children compared to healthy controls.

Serum IgE levels were higher in asthmatic vs. healthy controls. Vitamin D insufficiency correlated to higher s IgE and Hs-CRP levels which are markers of eosinophilic inflammation. Vitamin D, a member of the secosteroid family known to play a role in calcium or phosphate homeostasis and bone mineralization, is a potent immunological modulator, and is known to regulate cell proliferation and differentiation [27]. According to epidemiological evidence, there is a worldwide epidemic of vitamin D insufficiency or deficiency. Vitamin D deficiency has been linked to increased severity of bronchial asthma in children [28]. Deficiency in vitamin D3 is also reported to promote lung airway reactivity and increased doses of steroids to achieve control. Asthmatics with a deficiency in vitamin D may have a decline in lung volume and capacity and a diminished treatment response. Our findings may have important clinical implications, provided trace elements like serum zinc, selenium, and vitamin D3 may be biomarkers for poor asthma control. Indian studies are scarce and more so in children.

The limitations of the study were that we only analyzed serum levels of trace elements and not other tissues like hair and nail, which may reflect the long-term status of the trace elements. Confounders like eating habits and distribution of trace elements sources affecting their absorption would have affected the blood levels. We also did not do an intracellular magnesium assessment, which could be the reason for normal magnesium levels. Since asthma is a clinical diagnosis with multifactorial etiology, levels of trace elements and vitamin D3 can serve as a biomarker for the assessment of asthma. In conclusion, serum analysis of trace elements, zinc, selenium, and vitamin D3 should be considered in the workup of asthma. Low serum zinc and selenium were associated with poor asthma control in our study. An association exists between serum zinc and vitamin D3 levels, thus signifying their role in airway inflammation. Further studies on intervention to change the levels of trace elements underlying the structure-function changes in asthma, particularly in poor asthma control, are needed.

## Conclusions

Serum zinc, selenium, and vitamin D3 levels were very low in asthmatic children and were significantly low in the uncontrolled group. Since asthma is a clinical diagnosis with multifactorial etiology, trace elements levels, and vitamin D3 can add value and serve as a biomarker for their assessment. Inflammatory markers like HsCRP and serum IgE were also correlated with low vitamin D3 and zinc. We could confer our findings not on the mechanistic approach of each trace element, which was our limitation, but on the clinical data and asthma control in children, which has a translational value. Hence, further studies on intervention to change trace element levels underlying structure-function changes in asthma, particularly in poor asthma control, are needed.
